# O10.8. A PLURIPOTENTIAL AT RISK MENTAL STATE: INITIAL RESULTS

**DOI:** 10.1093/schbul/sby015.260

**Published:** 2018-04-01

**Authors:** Jessica A Hartmann, Barnaby Nelson, Spooner Rachael, G Paul Amminger, Andrew Chanen, Aswin Ratheesh, Christopher G Davey, Patrick D McGorry

**Affiliations:** Orygen, The National Centre of Excellence in Youth Mental Health

## Abstract

**Background:**

The development of the ultra-high risk (UHR) criteria for psychosis over 20 years ago created a new framework for research into subthreshold states in psychiatry. Since (i) early clinical phenotypes are overlapping and non-specific, and (ii) prevention research faces the challenge of achieving adequate statistical power when focusing on low incidence syndromes such as schizophrenia, we introduce an extension of the UHR approach in order to encompass trans-diagnostic targets. The ‘CHARMS’ (Clinical High at Risk Mental State) study aims to validate a set of pluripotential criteria to prospectively identify help-seeking young people at risk of developing a range of serious mental disorders.

**Methods:**

The CHARMS study is a cohort study of help-seeking young people aged 12–25 attending youth mental health services in Melbourne, Australia. New referrals meeting the CHARMS criteria are allocated to the CHARMS+ group; referrals under CHARMS threshold are allocated to CHARMS- (control) group. Transition status and clinical/functional outcomes are re-assessed at 6 and 12 months. The CHARMS criteria consist of subthreshold states for psychosis, mania, severe depression and borderline personality disorder. A range of clinical predictors, including anxiety, stress, sleep/circadian disturbance, and cognitive biases are being assessed as well.

**Results:**

To date, a sample of N=73 participants have been recruited: N=49 (67%) met CHARMS criteria (CHARMS+) at baseline with N=24 (33%) allocations to the control group (CHARMS-). Of these, N=48 participants have been followed up to 6 months and a sample of N=35 has been followed up to 12 months. At 6 months, 32% of the CHARMS+ group have transitioned to a full-threshold mental disorder which increased to 37% at 12 month follow-up. 0% of the CHARMS- control group has transitioned.

**Discussion:**

Our initial results indicate that the CHARMS criteria can be applied in the context of a youth mental health service and validly identify help-seeking young people at substantial risk of progressing to serious mental disorder over a short time frame (within 12 months). This study is the first to introduce and validate a set of clinical criteria to identify a broader ‘at risk’ patient population, and represents an important advance from the UHR for psychosis approach. It will foster understanding of risk factors and pathogenic mechanisms that drive the onset of severe mental disorder transdiagnostically and introduce a new case identification paradigm for the next generation of preventive intervention trials.

